# Performance enhancement method by using the probabilistic estimation for kidney tumor segmentation

**DOI:** 10.3389/fonc.2026.1764408

**Published:** 2026-03-27

**Authors:** Wonjoong Cheon, Meangee Kim, Mira Han, Se Byeong Lee, Dongho Shin, Young Kyung Lim, Jong Hwi Jeong, Yoonsun Chung, Haksoo Kim

**Affiliations:** 1 Department of Radiation Oncology, Seoul St. Mary’s Hospital, College of Medicine, The Catholic University of Korea, Seoul, Republic of Korea; 2 Proton Therapy Center, National Cancer Center, Goyang-si, Republic of Korea; 3 Department of Nuclear Engineering, Hanyang University, Seoul, Republic of Korea; 4 Department of Medical Research Collaborating Center, Seoul Metropolitan Government-Seoul National University Boramae Medical Center, Seoul, Republic of Korea

**Keywords:** deep learning, ensemble method, medical image segmentation, STAPLE algorithm, tumor segmentation

## Abstract

**Purpose:**

Ensemble methods can enhance segmentation performance, but their effectiveness depends on the integration strategy. We investigated whether the STAPLE algorithm’s probabilistic framework could effectively leverage model diversity from different loss functions to improve kidney tumor segmentation accuracy compared to individual models and conventional soft voting.

**Methods:**

We utilized CT scans from 210 patients in the KiTS19 dataset with expert-annotated kidney and tumor structures. Five model variants were developed using the nnU-Net framework: two 2D U-Nets and three 3D U-Nets, each trained with different hybrid loss functions (*L_CE+Dice_*, *L_TopK+Dice_*, *L_CE+GDice_*). Five approaches were compared: individual 2D U-Net, individual 3D U-Net, majority voting ensemble, soft voting ensemble, and STAPLE ensemble. Models underwent 5-fold cross-validation, and performance was evaluated using DSC, JI, HD95, precision, and recall on 63 test patients. Statistical significance was assessed using Wilcoxon signed-rank tests with Benjamini–Hochberg correction. Generalizability was evaluated on liver tumor segmentation using the LiTS17 dataset.

**Results:**

In KiTS19 tumor segmentation, individual model DSCs ranged from 0.64 ± 0.27 (2D models) to 0.70 ± 0.24 (3D models). Majority voting achieved DSC of 0.70 ± 0.27 and soft voting achieved 0.71 ± 0.26, while STAPLE reached 0.74 ± 0.23 (adjusted p<0.05). JI improved from 0.53-0.59 (individual models) to 0.63 ± 0.24 (STAPLE). HD95 decreased to 11.81 ± 13.43 with STAPLE. Precision and recall reached 0.88 ± 0.20 and 0.72 ± 0.24, respectively. In LiTS17 liver tumor segmentation, STAPLE similarly outperformed soft voting (DSC: 0.76 ± 0.10 vs. 0.71 ± 0.18, adjusted p<0.05).

**Conclusions:**

The STAPLE algorithm achieved superior performance in primary segmentation metrics compared to individual models, majority voting, and soft voting (STAPLE > soft voting > majority voting), demonstrating the benefits of probabilistic ensemble methods for kidney tumor segmentation. Stratified analysis revealed that STAPLE’s advantage was most pronounced for medium-sized tumors, where performance variability was reduced by 45%. The approach showed consistent effectiveness in liver tumor segmentation, suggesting potential for broader clinical applications.

## 1 Introduction

Through the advancement of imaging modality equipped with medical linear accelerators and the incorporation of deep-learning based algorithms, recent radiation therapy has been able to accommodate day-to-day anatomical variations of patients: adaptive radiation therapy (1, 2).

Specifically, in the contouring procedures, the deep-learning-based auto-contouring utilizing the U-Net architecture (3) has significantly reduced manual contouring time (4, 5). Zabel et al. reported that this integration decreases the initial contouring time for the rectum and bladder from 10.9 to 1.4 min, while the review and editing times by radiation oncologists average 4.1 and 4.7 min, respectively (6). Byun et al. demonstrated the efficacy of the auto-contouring technique in breast radiation therapy for nine organs-at-risk (OARs), reducing the time required to create clinically acceptable contours by 84% (7). Moreover, deep-learning-based auto-contouring has improved accuracy, reduced contouring time, and enhanced overall clinical efficiency in treating various cancers, including prostate, cervical, and head and neck cancers (8–10).

Although deep learning-based auto-contouring shows high performance and is applicable in clinical procedures for OAR segmentation, it remains a challenge in tasks related to tumor or target segmentation. Perslev et al. demonstrated that while the Dice similarity coefficients (DSC) for the liver, hippocampus, and spleen were high (approximately 0.94, 0.89, and 0.95, respectively), the coefficients for different brain tumors —edema, non-enhancing tumor, and enhancing tumor—were significantly lower (0.70, 0.43, and 0.67, respectively). For delineating the tumor of liver, lung, pancreas, and colon cancers, the DSC values were approximately 0.57, 0.59, 0.25, and 0.28, respectively, using the same single-model approach (11). A review of 21 studies focusing on kidney and kidney tumor segmentation revealed that the average DSC for kidney segmentation was approximately 0.96 ± 0.03, while for kidney tumors it was around 0.73 ± 0.15, highlighting the challenges in tumor segmentation (12).

To enhance the segmentation accuracy of deep-learning based model, recent researches have focused on increasing (1) the number of parameters in a model, (2) the dimensionality of the input layer, and (3) employing ensemble strategies. The number of parameters in a model is crucial for its performance. Generally, models with more parameters achieve higher accuracy. However, as the number of parameters increases, performance improvement tends to plateau, indicating that the relationship between model size and performance is not linear (13).

Hu et al. compared the segmentation accuracy among two-dimensional (2D), 2.5D, and 3D U-Net for nasopharyngeal carcinoma on magnetic resonance (MR) images. Notably, the 2.5D U-Net integrated results from three distinct models, each trained on images from orthogonal planes. Unlike its 2D and 2.5D counterparts, the 3D U-Net inherently processes inputs and outputs in three dimensions. According to their data, the segmentation performance improved from 2D to 2.5D to 3D with the processing time increasing accordingly (14). Vu et al. proposed a pseudo-3D method. This method utilizes a batch of multi-slice CT images where adjacent slices serve as additional channels to the central slice, culminating in a predicted binary mask for the central slice alone. This approach is more computationally efficient than traditional 3D U-Net and superior in performance to conventional 2D U-Nets (15).

Ensemble methods combine predictions from multiple models to improve segmentation accuracy (16, 17). To maximize ensemble effectiveness, model diversity can be introduced through various strategies: dataset shift (18), Monte-Carlo dropout (19), change of model hyperparameters (20, 21), or changes in the loss function (22). This diversity allows models to capture complementary features and compensate for individual weaknesses, thereby reducing overall prediction errors (23). Li et al. demonstrated that loss function diversity is an effective strategy for inducing model diversity in CT-based medical image segmentation (24).

Given diverse model outputs, the choice of fusion strategy determines how effectively this diversity translates into improved performance. Common approaches include majority voting, which assigns each voxel to the most frequently predicted class, and soft voting, which averages predicted probabilities across models. More advanced strategies include learnable ensemble methods that optimize model-specific weights through additional training (24), and probabilistic methods such as the Simultaneous Truth and Performance Level Estimation (STAPLE) algorithm (25), which iteratively estimates each model’s reliability through an expectation-maximization framework. Among these, STAPLE offers a distinct practical advantage: it operates entirely as a post-processing step on existing model outputs, requiring no additional training or architectural modifications, and is thus fully compatible with self-configuring frameworks such as nnU-Net. Recent studies have demonstrated the effectiveness of STAPLE as an ensemble strategy: Henderson et al. showed that STAPLE significantly improved head and neck OAR auto-segmentation across models trained with varying dataset sizes (26), and Sumon et al. employed STAPLE to fuse top-performing models for prostate gland segmentation, achieving improved delineation of zonal anatomy (27). However, these studies primarily addressed organs-at-risk or glandular structures with relatively consistent morphology, and induced model diversity through dataset size or architectural variations rather than loss function design.

**Figure 1:**
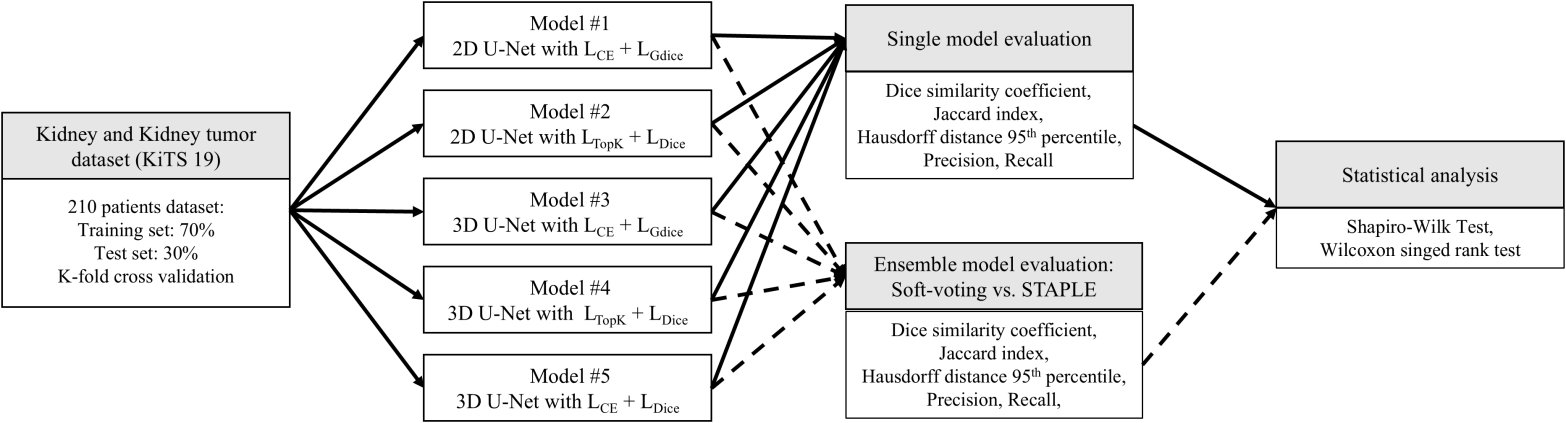
Workflow for assessing segmentation performances for an individual deep-learning model and ensemble methods for kidney tumor. *L_CE_*, cross-entropy loss; *L_TopK_*, Top-K loss; *L_Dice_*, dice loss; *L_GDice_*, generalized dice loss.

This study investigates the potential for performance enhancement in kidney tumor segmentation by inducing model diversity through variations in loss functions and implementing STAPLE as an ensemble method (**Figure 1**). Unlike prior STAPLE applications that targeted OAR or glandular structures (26, 27), we focus on tumor segmentation—a more challenging task due to irregular morphology, indistinct boundaries, and severe class imbalance—and systematically compare STAPLE against both soft voting and majority voting with rigorous statistical correction. To our knowledge, the combination of loss function-based model diversity with STAPLE for tumor segmentation has not been previously explored.

## 2 Materials and methods

### 2.1 Dataset

The kidney and kidney tumor segmentation 2019 (KiTS19) dataset is publicly available at https://github.com/neheller/kits19. This dataset includes patients who underwent partial or radical nephrectomy for one or more renal tumors suspected of malignancy at the University of Minnesota Medical Center between 2010 and 2018. The dataset was constructed through a retrospective review with a data collection protocol for 554 patients, ultimately verifying a dataset for 300 patients. Of these, the data for 210 patients are publicly available on the website. Among the 210 patient datasets, 147 were assigned to the training set (70%) and the remaining 63 were assigned to the test set (30%). The training set was used for 5-fold cross-validation, where each fold used approximately 80% of the training data (118 patients) for training and 20% (29 patients) for validation. No additional exclusion criteria were applied; all 210 publicly available cases were included.

The KiTS19 dataset provides semantic segmentations with two classes: kidney and tumor. No separate cyst label is provided; cysts were semantically included within the kidney class. Our task was configured as multi-class segmentation (background, kidney, and tumor), utilizing the entire CT volume without regional limitations. CT images and ground truth labels are provided in anonymized NIfTI format. The dataset exhibits considerable imaging variability: in-plane resolution ranged from 0.44 to 1.04 mm (mean: 0.80 ± 0.11 mm), and slice thickness from 0.50 to 5.00 mm (mean: 3.16 ± 1.74 mm). Each case contains contrast-enhanced CT scans with a single consensus ground truth segmentation mask. Detailed patient characteristics are described in the original publication (28).

### 2.2 Deep-learning model: nnU-Net

Ronneberger et al. introduced the U-Net for medical image segmentation. The standard U-Net is based on a convolutional neural network (CNN) and comprises an encoder and a decoder. Since its introduction, numerous U-Net variants have been proposed (29–31). However, to achieve high performance on an end user’s data, fine-tuning of the model’s hyperparameters is essential. Without proper fine-tuning, performance degradation on the end user’s data is inevitable, even if the model’s effectiveness has been validated by other research and datasets.

To minimize the model’s performance dependence affected by the user’s expertise, Isensee et al. proposed the no-new U-Net (nnU-Net) framework (32). The nnU-Net operates using a data-driven AutoML method. It extracts a ‘data fingerprint’—the characteristic features of the data—and most hyperparameters are determined in a rule-based manner according to this fingerprint. ‘Fixed-parameters’ such as architecture templates, optimizer, learning rate, and augmentation methods are set to default values but are designed to be adjustable by the user. Recently, the nnUNet version 2 (nnUNet-v2) was released, showing no performance disparity with version 1. The nnUNet-v2 framework officially provides various architecture templates: 2D U-Net, 3D full-resolution U-Net, 3D low-resolution U-Net, and 3D cascade U-Net. Therefore, in this study, we adopted nnUNet-v2 as the baseline framework to conduct experiments independent of model-specific tuning.

### 2.3 Loss functions

In an end-to-end trained deep-learning model, the loss function employed for the optimization process significantly influences its characteristics. In segmentation tasks, loss functions can be categorized into two types: (1) distribution-based loss functions and (2) region-based loss functions.

We aimed to enhance model diversity by employing cross-entropy loss (*L_CE_*), Dice loss (*L_Dice_*), and variations of loss functions tailored to address class imbalance.

#### 2.3.1 Distribution-based loss functions

Distribution-based loss functions compare the predicted probability distributions of class labels with the actual distributions in the ground truth. *L_CE_* is defined as Equation 1:

(1) $$L_{CE}=-\frac{1}{N}\sum_{c=1}^{C}\sum_{i=1}^{N}{ɡ}_{i}^{c}\log s_{i}^{c}$$

where *N* is the total number of pixels, *C* represents the number of classes, $g_{i}^{c}$ denotes the ground truth probability of pixel *i* being of class *c* and $s_{i}^{c}$ is the predicted probability for pixel *i* of class *c*. A specialized adaptation of the cross-entropy loss is the Top-K Loss (*L_TopK_*), which concentrates on the *K%* most challenging pixels, as defined in Equation 2:

(2) $$L_{TopK}\;=\;\frac{1}{N}\sum_{c=1}^{C}\sum_{i\in K}{ɡ}_{i}^{c}\;\log\;s_{i}^{c},$$

where *K* represents the set of pixels corresponding to the top *K%* of errors. This approach effectively allows the network to focus on hard samples during training, prioritizing areas of the image where the model’s performance is lacking.

#### 2.3.2 Region-based loss functions

Region-based loss functions are designed to optimize segmentation quality directly by comparing the spatial overlap between predictions and the ground truth. Among these, *L_Dice_* is a prominent example, aimed at maximizing the DSC, and is defined in Equation 3:

(3) $$L_{Dice}=1-2\frac{\sum_{c=1}^{C}\sum_{i=1}^{N}{ɡ}_{i}^{c}s_{i}^{c}}{\sum_{c=1}^{C}\sum_{i=1}^{N}{ɡ}_{i}^{c}+\sum_{c=1}^{C}\sum_{i=1}^{N}s_{i}^{c}}$$

where *N* is the total number of pixels, *C* represents the number of classes, $g_{i}^{c}$ denotes the ground truth indicator for pixel *i* being of class *c*, and $s_{i}^{c}$ is the predicted probability of pixel *i* for class *c*.

Generalized Dice Loss (*L_GDice_*) extends DL for multi-class segmentation tasks by incorporating class-specific weights​ which are inversely proportional to the frequency of each class, thereby addressing class imbalance. It is defined as shown in Equation 4:

(4) $$L_{GDice}=1-2\frac{\sum_{c=1}^{C}w_{c}\sum_{i=1}^{N}{ɡ}_{i}^{c}s_{i}^{c}}{\sum_{c=1}^{C}w_{c}\sum_{i=1}^{N}\left({ɡ}_{i}^{c}+s_{i}^{c}\right)}$$

where *w_c_*​ is the weight assigned to each class *c*, designed to be inversely proportional to the class’s frequency in the dataset, enhancing the model’s sensitivity to less represented classes and contributing to a more balanced training process.

#### 2.3.3 Hybrid loss functions

To ensure model diversity, we implemented hybrid loss functions such as *L_CE_* + *L_Dice_* (Equation 5), *L_TopK_* + *L_Dice_* (Equation 6), and *L_CE_* + *L_GDice_* (Equation 7). These are represented as follows:

(5) $$L_{CE+Dice}=L_{CE}+L_{Dice}$$

(6) $$L_{TopK+Dice}=L_{TopK}+L_{Dice}$$

(7) $$L_{CE+GDice}=L_{CE}+L_{GDice}$$

These hybrid loss functions offer a balanced approach to penalizing false positives and false negatives, thus enhancing pixel-wise accuracy and the overall quality of segmentation. Combining these functions can significantly influence the model’s performance. For example, incorporating *L_CE_* with *L_Dice_* can improve the model’s ability to handle imbalanced data, optimizing both sensitivity and specificity. Meanwhile, combining *L_TopK_* with *L_Dice_* might focus the model’s learning efforts on the most challenging pixels, potentially increasing precision in areas with high variability.

### 2.4 Training strategy

To maximize the effectiveness of the ensemble, it is crucial to ensure model diversity. Within the nnU-Net framework, five different single models were trained with hybrid loss functions. Both the 2D and 3D full-resolution U-Nets were used as the single models. The five combinations of single models with their respective loss functions are as follows:

Model 1 (M1): 2D U-Net with $L_{CE+GDice}$Model 2 (M2): 2D U-Net with $L_{TopK\left(K=10\%\right)+Dice}$Model 3 (M3): 3D U-Net with $L_{CE+GDice}$Model 4 (M4): 3D U-Net with $L_{TopK\left(K=10\%\right)+Dice}$Model 5 (M5): 3D U-Net with $L_{CE+Dice}$All U-Net architectures underwent 5-fold cross-validation using the 147 training cases. Based on automated data fingerprinting, the nnU-Net framework configured: resampling to 1.0×1.0×1.0 mm³ isotropic spacing, intensity windowing to [-79, 304] HU, z-score normalization, patch sizes of 512×512 (2D model) and 288×272×32 (3D model), and mini-batch sizes of 12 and 2, respectively. Data augmentation techniques such as rotation, flipping, and gamma intensity transformation were implemented to mitigate overfitting. The model parameters were saved when the validation set loss value was minimized. Training epochs were set to 5000, while other hyperparameters remained at their default settings, determined through the data fingerprint acquisition process in the nnUNet-v2 framework.

### 2.5 Ensemble strategies

Each model variant (M1–M5) was trained using 5-fold cross-validation. Our ensemble process operated at two levels. At the fold level, predictions from the five cross-validation folds were combined using the soft voting method provided by the nnU-Net framework, which averages softmax probabilities to produce a single prediction per model variant (V_model1_, V_model2_, V_model3_, V_model4_, V_model5_). At the model level, the outputs from models trained with different loss functions were combined using three ensemble strategies: soft voting, majority voting, and STAPLE.

For soft voting (V_soft voting_), softmax probabilities were averaged across models. We evaluated pairwise combinations ({M1, M2}, {M1, M3}, {M1, M4}, {M2, M3}, {M2, M4}, {M3, M4}) and the full combination ({M1, M2, M3, M4, M5}).

For majority voting (V_majority_), binary segmentation masks from each model were combined by assigning each voxel to the class predicted by the majority of models. A voxel was labeled as tumor if more than half of the models predicted it as tumor. Majority voting was evaluated using the full combination of all five models ({M1, M2, M3, M4, M5}).

For STAPLE ensemble (V_STAPLE_), we integrated binary segmentation masks from all five models ({M1, M2, M3, M4, M5}) through probabilistic fusion. The algorithm iteratively estimates sensitivity and specificity parameters for each model, using these to compute weighted contributions to the final segmentation. Initial sensitivity and specificity were set to 0.9999 for all models, with a convergence tolerance of 1×10^-4^ and maximum iterations of 100. The algorithm produces probabilistic maps that require thresholding for final binary segmentation. Optimal thresholds were determined through grid search on the validation set, resulting in 0.2 for tumor regions in KiTS19, selected to maximize DSC while maintaining balanced precision and recall.

### 2.6 Evaluation

To evaluate the accuracy of the tumor segmentation predictions, we utilized metrics such as the DSC, Jaccard index (JI), and the 95^th^ percentile Hausdorff Distance (HD95). The DSC and JI quantified the spatial overlap between each model’s segmentation result and the ground truth. The formulas for calculating DSC and JI are as follows in Equations 8 and 9:

(8) $$DSC=\;\frac{2\left|A\cap B\right|}{\left|A\right|+\left|B\right|}\text{ or }\frac{2TP}{\left(TP+FN\right)+\left(TP+FP\right)}$$

(9) $$\;JI=\;\frac{\left|A\cap B\right|}{\left|A\cup B\right|}\text{ or }\frac{TP}{TP+FP+FN}$$

where A represents the ground truth and B denotes the prediction. Here, True Positive (TP) occurs when both the ground truth and prediction correctly indicate a tumor presence, True Negative (TN) when both indicate its absence, False Positive (FP) when only the prediction indicates a tumor presence, and False Negative (FN) when only the ground truth does.

The HD95 metric, measured in millimeters (mm), is calculated only when both the ground truth and the predicted mask indicate the presence of a tumor, focusing on the 95^th^ percentile of distances between the boundaries of these regions.

Furthermore, precision and recall were employed to measure the model’s performance, derived from a confusion matrix that enables pixel-by-pixel comparison between the ground truth and the predicted output. Precision and recall, defined based on the confusion matrix, are as follows in Equations 10 and 11:

(10) $$Precision=\;\frac{TP}{\left(TP+FP\right)}$$

(11) $$Recall=\;\frac{TP}{\left(TP+FN\right)}$$

Sensitivity is equivalent to recall as defined in Equation 11. Voxel-level specificity exceeded 0.99 for all methods due to the overwhelming proportion of background voxels in volumetric segmentation, rendering it uninformative for method comparison; we therefore report precision (positive predictive value) as a more discriminative complement to recall.

These metrics were applied to assess the accuracy of five different models and to evaluate the effectiveness of soft voting, majority voting, and the STAPLE algorithm as ensemble methods.

To establish the statistical significance of differences between models, we conducted normality tests using the Shapiro-Wilk test. The null hypothesis (H_0_: normality) was rejected, leading us to perform the Wilcoxon signed-rank test, a non-parametric alternative. Pairwise comparisons were conducted among three method categories: STAPLE ensemble, soft voting ensemble, and individual models (M1–M5), yielding 11 comparisons per metric. Majority voting was reported descriptively as an additional reference method. To account for multiple comparisons, the Benjamini-Hochberg procedure was applied within each metric family (DSC, JI, HD95, Precision, and Recall) to control the false discovery rate at α = 0.05. Statistical significance was determined based on adjusted p-values. The analysis was performed using SAS software (version 9.4; SAS Institute, Cary, NC, USA), and the figures were created using R version 4.3.1 (R Foundation for Statistical Computing, Vienna, Austria).

### 2.7 Generalizability assessment

To evaluate the transferability of our STAPLE-based ensemble approach to different organ systems, we applied the same methodology to liver tumor segmentation using the Liver Tumor Segmentation 2017 (LiTS17) dataset, which consists of contrast-enhanced abdominal CT scans from 131 patients with primary liver cancers or metastatic liver tumors collected from seven hospitals and research institutions. Among the 131 patient datasets, 92 were assigned to the training set (70%) and the remaining 39 were assigned to the test set (30%). The training set was used for 5-fold cross-validation, where each fold used approximately 80% of the training data (73 patients) for training and 20% (18 patients) for validation. No additional exclusion criteria were applied; all 131 cases were included.

The LiTS17 dataset provides semantic segmentations with two classes: liver and tumor. Our task was configured as multi-class segmentation (background, liver, and tumor), utilizing the entire CT volume. The dataset exhibits similar imaging variability to KiTS19: in-plane resolution ranged from 0.56 to 1.00 mm (mean: 0.79 ± 0.11 mm), and slice thickness from 0.70 to 5.00 mm (mean: 1.42 ± 1.08 mm). Each case contains contrast-enhanced CT scans with a ground truth segmentation mask verified by experienced radiologists (33).

The nnU-Net framework automatically configured all preprocessing and training parameters through its data fingerprinting process: images were resampled to 0.767×0.767×1.0 mm³, intensity windowed to [-20.0, 201.0] HU with z-score normalization, using patch sizes of 512×512 (2D model) and 128×128×128 (3D model) with mini-batch sizes of 12 and 2, respectively. We maintained the same five model variants (M1–M5) and compared soft voting, majority voting, and STAPLE ensemble methods using identical parameters as described in Section 2.5. Performance evaluation utilized the same metrics (DSC, JI, HD95, precision, recall) on the independent test set.

## 3 Results

### 3.1 A single model with various loss functions

**Figure 2:**
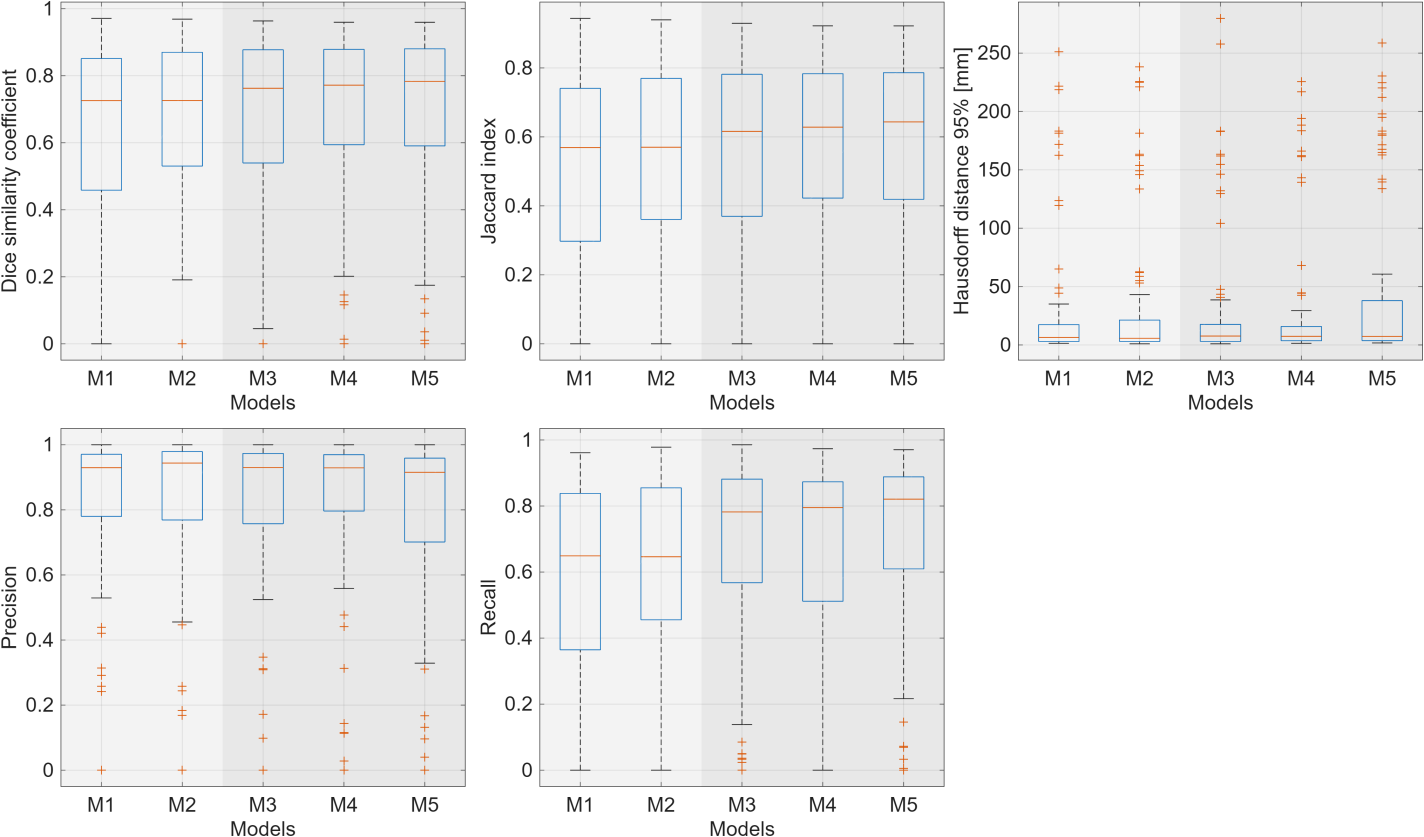
Comparison of performance metrics across five single models (M1-M5).

Individual model performances were evaluated as a baseline for comparison with ensemble techniques. All results presented below are based on the evaluation of the 63 test patients. The performance of these models is summarized in **Table 1** and visualized in **Figure 2**.

**Table 1:** Comparative performance metrics of 2D and 3D U-Net models under various loss functions for kidney tumor segmentation.

Metric	2D (M1) $L_{CE+GDice}$	2D (M2) $L_{TopK\left(K=10\%\right)+Dice}$	3D (M3) $L_{CE+GDice}$	3D (M4) $L_{TopK\left(K=10\%\right)+Dice}$	3D (M5) $L_{CE+Dice}$
DSC	0.64 ± 0.27	0.65 ± 0.26	0.68 ± 0.27	0.68 ± 0.27	0.70 ± 0.24
JI	0.52 ± 0.27	0.53 ± 0.26	0.56 ± 0.27	0.57 ± 0.27	0.59 ± 0.25
HD95 (mm)	16.72 ± 13.92	14.93 ± 14.50	17.81 ± 24.09	18.37 ± 30.44	21.69 ± 39.02
Precision	0.82 ± 0.23	0.82 ± 0.25	0.82 ± 0.24	0.81 ± 0.25	0.80 ± 0.25
Recall	0.59 ± 0.29	0.61 ± 0.28	0.67 ± 0.29	0.67 ± 0.29	0.71 ± 0.26

DSC, Dice similarity coefficient; JI, Jaccard index; HD95, Hausdorff distance 95^th^ percentile.

Specifically, the DSC for 2D U-Nets ranged from 0.64 to 0.65, while 3D U-Nets achieved higher values ranging from 0.68 to 0.70, demonstrating the benefit of leveraging volumetric context. The JI showed a similar trend, ranging from 0.52 to 0.53 for 2D models and from 0.56 to 0.59 for 3D models. The HD95 ranged from 14.93 to 16.72 mm for 2D models and from 17.81 to 21.69 mm for 3D models. Precision was comparable across both architectures, ranging from 0.80 to 0.82 for all models. However, recall differed more substantially, ranging from 0.59 to 0.61 for 2D models and from 0.67 to 0.71 for 3D models, suggesting more comprehensive tumor detection by the 3D U-Nets. In pairwise comparisons, the differences in recall between 2D and 3D models were statistically significant.

### 3.2 Ensemble methods: majority voting, soft voting, and STAPLE algorithm

To quantitatively analyze the effects of ensemble methods, soft voting, majority voting, and the STAPLE algorithm were employed to integrate outputs from various model combinations, using the parameters and thresholds described in Section 2.5.

**Table 2** summarizes the performance metrics, including DSC, JI, HD95, precision, and recall, for the soft voting ensemble methods across pairwise and full model combinations. Most evaluation metrics showed improvements compared to the individual models. Among soft voting combinations, integrating all models (M1, M2, M3, M4, M5) achieved the highest DSC of 0.71 ± 0.26 and recall of 0.68 ± 0.28, while pairwise combinations showed varying degrees of improvement depending on the model pairs. However, soft voting did not significantly outperform the best individual model (M5) in overlap-based metrics (DSC: adjusted p = 0.112; JI: adjusted p = 0.104), although significant improvements were observed for HD95 (adjusted p = 0.009) and precision (adjusted p< 0.001).

**Table 2:** Comparative performance metrics of ensemble methods for kidney tumor segmentation.

Metric	Soft voting							Majority voting	STAPLE
	M1, M2	M1, M3	M1, M4	M2, M3	M2, M4	M3, M4	M1–M5	M1–M5	M1–M5
DSC	0.65 ± 0.26	0.69 ± 0.26	0.69 ± 0.26	0.70 ± 0.27	0.69 ± 0.27	0.69 ± 0.27	0.71 ± 0.26	0.70 ± 0.27	0.74 ± 0.23^*†^
JI	0.54 ± 0.26	0.58 ± 0.27	0.57 ± 0.26	0.59 ± 0.27	0.59 ± 0.27	0.58 ± 0.27	0.60 ± 0.27	0.60 ± 0.27	0.63 ± 0.24^*†^
HD95 (mm)	14.86 ± 12.86	12.85 ± 11.34	13.86 ± 11.26	12.28 ± 11.20	13.39 ± 12.26	18.24 ± 23.00	13.42 ± 15.71	21.47 ± 46.62	11.81 ± 13.43^†^
Precision	0.85 ± 0.21	0.88 ± 0.20	0.87 ± 0.22	0.84 ± 0.22	0.85 ± 0.26	0.83 ± 0.25	0.86 ± 0.23	0.85 ± 0.24	0.88 ± 0.20^*†^
Recall	0.60 ± 0.28	0.63 ± 0.28	0.62 ± 0.28	0.65 ± 0.29	0.64 ± 0.28	0.67 ± 0.29	0.68 ± 0.28	0.68 ± 0.28	0.72 ± 0.24^***^

DSC, Dice similarity coefficient; JI, Jaccard index; HD95, Hausdorff distance 95^th^ percentile.

*p< 0.05, ***p< 0.001 vs. soft voting (M1–M5), Benjamini-Hochberg adjusted. † p< 0.05 vs. best individual model (M5), Benjamini-Hochberg adjusted.

See **Table 3** for detailed pairwise comparisons.

Majority voting using all five models (M1–M5) achieved a DSC of 0.70 ± 0.27 for kidney tumor segmentation, showing comparable performance to soft voting (DSC: 0.71 ± 0.26). While majority voting improved upon most individual models, its binary decision process—which does not account for prediction confidence—limited further gains compared to methods that leverage probability information.

The STAPLE ensemble of all five models achieved the best overall performance, with DSC of 0.74 ± 0.23, JI of 0.63 ± 0.24, HD95 of 11.81 ± 13.43, precision of 0.88 ± 0.20, and recall of 0.72 ± 0.24. Compared to individual models (M1–M4), STAPLE demonstrated highly significant improvements across all metrics (adjusted p< 0.01). Against the best individual model (M5), STAPLE achieved significant improvements in DSC (adjusted p = 0.037), JI (adjusted p = 0.026), HD95 (adjusted p< 0.001), and precision (adjusted p< 0.001), while recall did not differ significantly (adjusted p = 0.484), consistent with M5 already exhibiting the highest recall among individual models. Compared to soft voting, STAPLE showed significant improvements in recall (adjusted p< 0.001) and precision (adjusted p = 0.044), with DSC (adjusted p = 0.046) and JI (adjusted p = 0.047) reaching statistical significance at narrow margins after Benjamini–Hochberg correction. HD95 did not differ significantly between the two ensemble methods (adjusted p = 0.652). Detailed pairwise comparisons with adjusted p-values are presented in **Table 3**.

**Table 3:** Benjamini–Hochberg adjusted p-values for pairwise comparisons of ensemble methods for kidney tumor segmentation.

Metric	STAPLE vs. Soft voting	STAPLE vs. M5	Soft voting vs. M5
DSC	0.046 *	0.037 *	0.112
JI	0.047 *	0.026 *	0.104
HD95	0.652	< 0.001 ***	0.009 **
Precision	0.044 *	< 0.001 ***	< 0.001 ***
Recall	< 0.001 ***	0.484	0.016 *

Benjamini–Hochberg adjusted p-values from Wilcoxon signed-rank tests.

Corrections were applied within each metric family (11 comparisons per metric).

*p< 0.05, **p< 0.01, ***p< 0.001.

**Figure 3:**
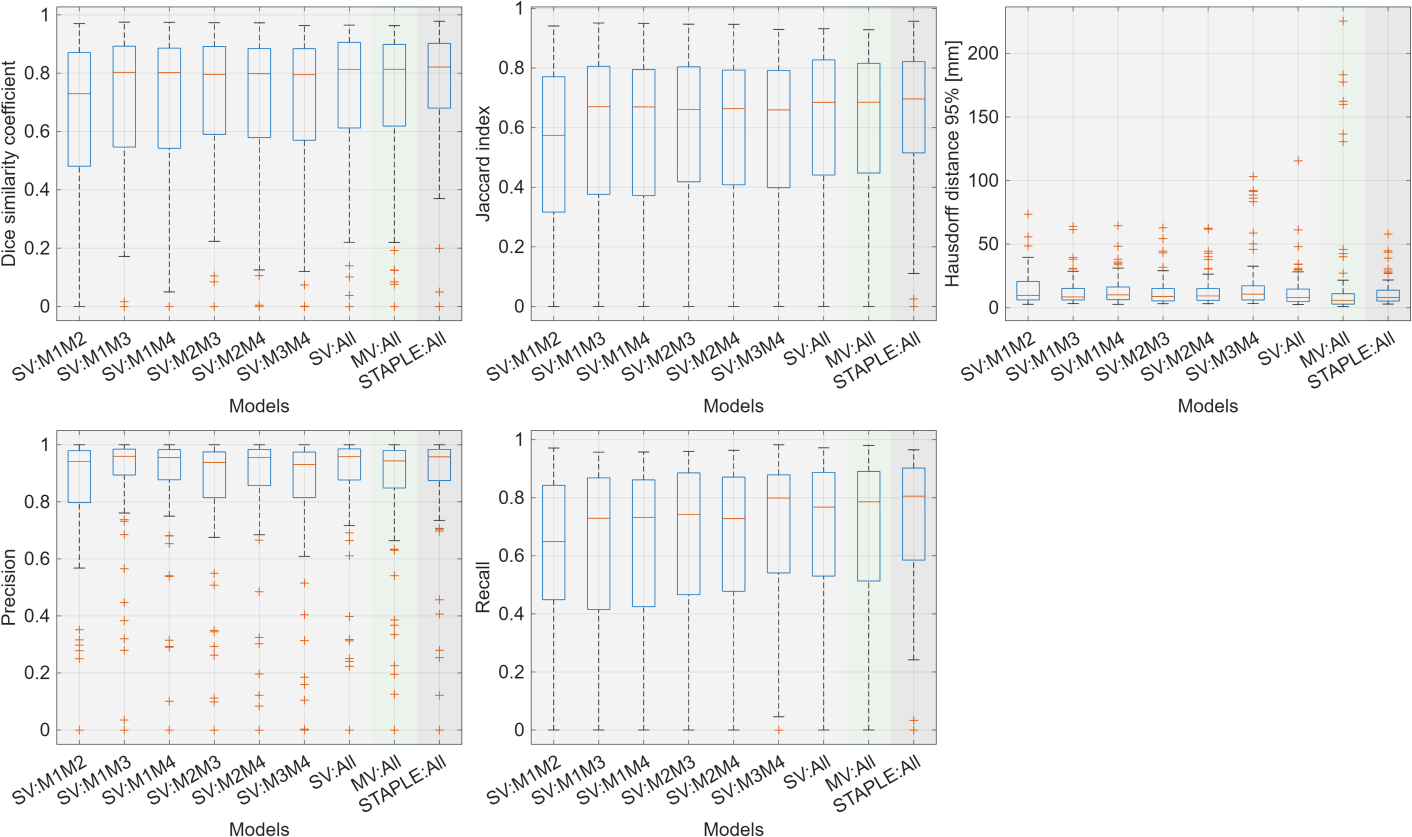
Comparison of performance metrics across ensemble methods: soft voting, majority voting, and STAPLE.

**Figure 4:**
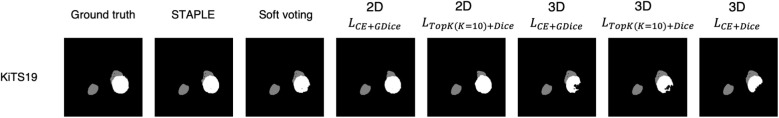
Qualitative comparison of kidney tumor segmentation results from individual models trained with different loss functions and ensemble methods.

Overall, the three ensemble strategies showed a clear performance hierarchy: STAPLE > soft voting > majority voting ≥ individual models. The superiority of STAPLE can be attributed to its probabilistic framework, which estimates each model’s reliability and weights contributions accordingly, rather than treating all models equally (majority voting) or averaging predictions without performance-based weighting (soft voting). Notably, while the mean performance differences between STAPLE and soft voting were modest, STAPLE demonstrated a consistent reduction in performance variance (e.g., DSC standard deviation decreased from 0.26 to 0.23), suggesting that its primary advantage lies in stabilizing predictions across morphologically heterogeneous cases. These comparisons are visualized in **Figure 3**. Representative segmentation examples demonstrating the visual quality differences between methods are shown in **Figure 4**.

### 3.3 Stratified analysis by tumor volume

To examine the relationship between tumor size and segmentation performance, the 63 test cases were stratified into tertiles based on ground truth tumor volume: Small (≤ 5,897 voxels, n = 21), Medium (5,898–21,784 voxels, n = 21), and Large (> 21,784 voxels, n = 21). **Table 4** summarizes DSC performance by size group.

**Table 4:** Stratified DSC performance by tumor volume for KiTS19 kidney tumor segmentation.

Methods	Small (n = 21) ≤ 5,897	Medium (n = 21) 5,898–21,784	Large (n = 21) > 21,784
Best Single (M5)	0.55 ± 0.28	0.73 ± 0.22	0.83 ± 0.11
Majority Voting	0.52 ± 0.30	0.75 ± 0.23	0.84 ± 0.11
Soft Voting	0.52 ± 0.31	0.77 ± 0.18	0.84 ± 0.11
STAPLE	0.56 ± 0.30	0.81 ± 0.12	0.85 ± 0.12

Performance was strongly size-dependent across all methods, with mean DSC increasing from 0.52–0.56 for small tumors to 0.83–0.85 for large tumors. STAPLE demonstrated its greatest advantage in the medium group, achieving DSC of 0.81 ± 0.12 compared to 0.73 ± 0.22 for the best individual model (M5), representing a 45% reduction in performance variability. For small tumors, no method achieved reliable performance, and five cases showed DSC below 0.3 across all methods, attributable to the inherent difficulty of segmenting small volumes. For large tumors, all methods performed well with limited room for improvement.

### 3.4 Generalizability assessment

Evaluation on the LiTS17 liver tumor dataset examined whether our STAPLE-based ensemble approach could transfer to different organ systems. To assess whether the STAPLE parameters optimized for kidney tumors generalize to a different tumor type, we applied the same parameters (initial sensitivity/specificity: 0.9999, convergence tolerance: 1×10^-4^) and threshold (0.2) from KiTS19 without additional optimization. The algorithm successfully converged for all liver tumor cases.

As shown in **Table 5**, the STAPLE ensemble (M1–M5) achieved DSC of 0.76 ± 0.10, a 7.0% improvement over soft voting (0.71 ± 0.18, adjusted p = 0.030). Majority voting achieved DSC of 0.72 ± 0.17, comparable to soft voting but lower than STAPLE, consistent with the performance hierarchy observed in KiTS19. This improvement magnitude was comparable to the 4.2% enhancement observed in KiTS19, suggesting consistent benefits across different organ systems. STAPLE also reduced performance variance, with DSC standard deviation decreasing from 0.15–0.23 (paired models) and 0.18 (soft voting) to 0.10 in the STAPLE ensemble. The JI improved from 0.58 ± 0.19 to 0.62 ± 0.13, while recall increased from 0.62 ± 0.23 to 0.70 ± 0.16, indicating better tumor detection. These improvements in DSC, JI, and recall were statistically significant compared to both soft voting and the best individual model (M4) (adjusted p< 0.05, **Table 6**). However, precision decreased from 0.94 ± 0.07 (soft voting) to 0.89 ± 0.14 (STAPLE, adjusted p = 0.003), reflecting a trade-off where STAPLE’s improved tumor detection came at the cost of slightly more false positives—a pattern consistent with the recall-precision balance observed in KiTS19.

**Table 5:** Comparative performance metrics of ensemble methods for liver tumor segmentation.

Metric	Soft voting							Majority voting	STAPLE
	M1, M2	M1, M3	M1, M4	M2, M3	M2, M4	M3, M4	M1–M5	M1–M5	M1–M5
DSC	0.66 ± 0.20	0.70 ± 0.22	0.69 ± 0.18	0.71 ± 0.17	0.70 ± 0.15	0.70 ± 0.23	0.71 ± 0.18	0.72 ± 0.17	0.76 ± 0.10^*†^
JI	0.52 ± 0.21	0.57 ± 0.21	0.56 ± 0.20	0.57 ± 0.19	0.56 ± 0.18	0.57 ± 0.21	0.58 ± 0.19	0.58 ± 0.18	0.62 ± 0.13^*†^
HD95 (mm)	17.55 ± 26.20	14.13 ± 25.10	13.24 ± 21.80	11.72 ± 21.06	14.65 ± 22.87	8.82 ± 8.57	6.98 ± 5.47	6.12 ± 5.67	11.03 ± 21.68
Precision	0.90 ± 0.16	0.86 ± 0.24	0.90 ± 0.16	0.90 ± 0.15	0.93 ± 0.09	0.92 ± 0.10	0.94 ± 0.07	0.94 ± 0.06	0.89 ± 0.14^**†^
Recall	0.56 ± 0.22	0.62 ± 0.25	0.59 ± 0.23	0.62 ± 0.22	0.60 ± 0.20	0.62 ± 0.26	0.62 ± 0.23	0.62 ± 0.22	0.70 ± 0.16^**†^

DSC, Dice similarity coefficient; JI, Jaccard index; HD95, Hausdorff distance 95^th^ percentile.

*p< 0.05, **p< 0.01 vs. soft voting (M1–M5), Benjamini–Hochberg adjusted. † p< 0.05 vs. best individual model (M4), Benjamini–Hochberg adjusted.

See **Table 6** for detailed pairwise comparisons.

**Table 6:** Benjamini–Hochberg adjusted p-values for pairwise comparisons of ensemble methods for liver tumor segmentation.

Metric	STAPLE vs. Soft voting	STAPLE vs. M4	Soft voting vs. M4
DSC	0.030*	0.013 *	0.626
JI	0.031*	0.017 *	0.626
HD95	0.227	0.301	0.363
Precision	0.003**	0.011*	0.124
Recall	0.005**	0.004**	0.761

Benjamini–Hochberg adjusted p-values from Wilcoxon signed-rank tests.

Corrections were applied within each metric family (11 comparisons per metric).

*p< 0.05, **p< 0.01, ***p< 0.001.

**Figure 5:**
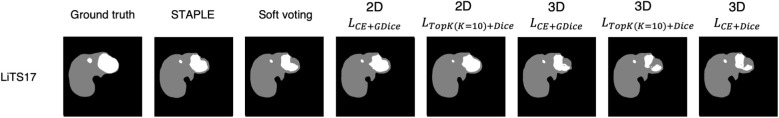
Qualitative comparison of liver tumor segmentation results from individual models trained with different loss functions and ensemble methods.

Among individual model pairs, M2–M3 combinations showed particularly strong baseline performance (DSC 0.71 ± 0.17), yet STAPLE integration of all models still provided meaningful improvements. Notably, soft voting did not significantly outperform the best individual model (M4) for any metric (adjusted p > 0.05, **Table 6**), whereas STAPLE achieved significant improvements over M4 in DSC, JI, precision, and recall (adjusted p< 0.05). While HD95 metrics did not differ significantly among ensemble methods, the primary segmentation quality indicators consistently favored STAPLE. These findings validate that our probabilistic ensemble approach maintains its effectiveness across different tumor types and imaging characteristics, supporting its potential for broader clinical applications. Visual comparison of segmentation results on the LiTS17 dataset is presented in **Figure 5**.

## 4 Discussion

This study demonstrates that the STAPLE algorithm improves tumor segmentation accuracy through probabilistic weighting of predictions from diverse models trained with different loss functions. Our approach achieved consistent improvements across both kidney and liver tumor segmentation tasks, supporting the effectiveness of probabilistic ensemble methods in medical imaging.

The STAPLE ensemble achieved DSC of 0.74 ± 0.23 for kidney tumors and 0.76 ± 0.10 for liver tumors, representing 4.2% and 7.0% improvements over soft voting ensemble method, respectively. Among the three ensemble strategies evaluated, a clear performance hierarchy emerged: STAPLE outperformed soft voting, which in turn outperformed majority voting (DSC: 0.70 ± 0.27 for kidney tumors). Majority voting, while improving upon individual models, was limited by its binary decision process that discards prediction confidence. Soft voting preserved probability information through averaging but treated all models equally regardless of their reliability. STAPLE’s superiority over both methods can be attributed to its iterative expectation-maximization process, which estimates each model’s sensitivity and specificity to weight contributions accordingly. Notably, in LiTS17, STAPLE’s improved recall (0.70 vs. 0.62) came at the cost of reduced precision (0.89 vs. 0.94, adjusted p = 0.003), reflecting a recall-precision trade-off consistent with STAPLE’s tendency to favor inclusive segmentation boundaries. This trade-off should be considered in clinical contexts where false positive minimization is prioritized, such as treatment planning near critical structures. This finding has practical implications, as soft voting is the default ensemble strategy in widely-used frameworks like nnU-Net. Our results suggest that STAPLE may offer particular advantages for challenging segmentation tasks such as tumor delineation where model uncertainty is high.

Our strategy of inducing model diversity through varied loss functions successfully generated complementary model behaviors in the KiTS19 dataset, as evidenced by statistically significant differences in recall metrics between 2D and 3D models. For kidney tumors, the 3D U-Net architectures generally outperformed their 2D counterparts (DSC: 0.68-0.70 vs 0.64-0.65), likely due to their ability to leverage volumetric context. However, the ensemble of both 2D and 3D models ultimately provided the best results, suggesting that architectural diversity further enhances ensemble performance.

A notable finding was STAPLE’s ability to reduce performance variance across both datasets. In kidney tumor segmentation, the standard deviation decreased from 0.26 (soft voting) to 0.23 (STAPLE), while liver tumor segmentation showed even greater stabilization (0.18 to 0.10). This variance reduction indicates more reliable and consistent predictions, which is crucial for clinical applications where unpredictable failures could impact treatment planning.

The higher variance observed in KiTS19 STAPLE ensemble (DSC SD: 0.23) compared to LiTS17 (DSC SD: 0.10) can be attributed to differences in tumor morphological heterogeneity. Analysis of tumor solidity (ratio of tumor area to convex hull area) revealed that KiTS19 exhibited a highly skewed distribution (skewness = -3.78), with 75.2% of cases showing regular morphology (solidity≥0.9) while 3.3% presented extremely irregular boundaries (solidity<0.5). This “easy majority with hard minority” pattern resulted in high performance for most cases but substantial failures for morphologically atypical tumors, thereby increasing overall variance. In contrast, LiTS17 showed more uniform morphological complexity (68.2% with solidity<0.5), leading to consistently moderate performance across cases and lower variance. These findings suggest that dataset-specific tumor heterogeneity should be considered when interpreting segmentation performance metrics.

The stratified analysis by tumor volume further supports this interpretation. STAPLE’s primary advantage was concentrated in medium-sized tumors, where individual models produced heterogeneous but partially correct predictions that STAPLE’s probabilistic weighting could effectively reconcile. In contrast, small tumors presented a fundamental detection challenge that no ensemble method could overcome, as evidenced by consistently poor performance across all approaches. Large tumors, being well-represented in training data and morphologically regular, were reliably segmented by all methods with minimal benefit from ensembling. These findings suggest that STAPLE is most valuable in clinical scenarios involving intermediate-complexity cases, and that alternative strategies such as dedicated small-lesion detection networks may be needed to address the persistent challenge of small tumor segmentation.

Application to liver tumors using the same experimental protocol showed that our approach maintains effectiveness across different organ systems. Despite distinct imaging characteristics between kidney and liver tumors—including variations in contrast enhancement patterns, tumor heterogeneity, and anatomical boundaries—STAPLE achieved consistent improvements over baseline methods. Notably, soft voting did not significantly outperform the best individual model (M4) for any metric in LiTS17 (adjusted p > 0.05, **Table 6**), whereas STAPLE achieved significant improvements over M4 in DSC, JI, precision, and recall, reinforcing STAPLE’s unique value as an ensemble strategy beyond simple probability averaging. This cross-organ evaluation suggests broader applicability for the probabilistic ensemble approach in medical image segmentation.

From a methodological perspective, our use of the nnU-Net framework ensures reproducibility and eliminates potential bias from manual hyperparameter tuning. The framework’s automated configuration through data fingerprinting allowed us to focus on validating the ensemble methodology rather than optimizing individual model architectures. While we deliberately used standard configurations rather than pursuing state-of-the-art performance, the consistent improvements achieved by STAPLE suggest that similar proportional gains (4-7%) could be expected when applied to more advanced architectures, potentially pushing SOTA methods even higher.

Our comparison encompassed three representative ensemble strategies with distinct fusion mechanisms: majority voting (binary decision without confidence), soft voting (equal-weight probability averaging), and STAPLE (performance-based probabilistic weighting). Our findings are consistent with prior reports demonstrating the effectiveness of STAPLE as an ensemble strategy for medical image segmentation. Henderson et al. showed that ensemble methods including STAPLE significantly improved head and neck OAR auto-segmentation across models trained with varying dataset sizes (26), and Sumon et al. employed STAPLE to fuse top-performing models for multiclass prostate gland segmentation, achieving improved delineation of zonal anatomy (27). However, these studies primarily addressed organs-at-risk or glandular structures with relatively consistent morphology and induced model diversity through dataset size or architectural variations. Our study extends these findings to tumor segmentation, which presents fundamentally different challenges due to irregular morphology, indistinct boundaries, and severe class imbalance, and demonstrates that STAPLE can effectively leverage diversity induced specifically through loss function variation.

Recent work by Li et al. proposed a learnable ensemble approach that dynamically combines models trained with different loss functions using optimized weights, achieving 2–7% DSC improvements for CT organ-at-risk segmentation (24). While their learnable approach offers the advantage of adaptive weight optimization, it requires approximately 3× parameter increase and an additional training phase. In contrast, our STAPLE-based approach achieved comparable improvements (4.2–7.0%) without requiring additional model training or significant computational overhead. Despite the more demanding conditions of tumor segmentation compared to the organ-at-risk targets evaluated by Li et al. (24), Henderson et al. (26), and Sumon et al. (27), our approach achieved similar or greater magnitudes of improvement, suggesting that STAPLE’s probabilistic weighting may be particularly effective when model uncertainty is high. While more sophisticated ensemble strategies such as uncertainty-weighted methods or learnable fusion layers may offer marginal additional gains, they introduce complexity that may limit clinical applicability. Similarly, several variants of the STAPLE algorithm have been proposed, including Local MAP STAPLE, which incorporates spatially varying performance parameters (35), and SIMPLE (Selective and Iterative Method for Performance Level Estimation), which iteratively excludes poorly performing segmentations (36). Since the primary aim of our study was to evaluate the effectiveness of loss function-driven model diversity with probabilistic ensemble fusion rather than to optimize the fusion algorithm itself, and standard STAPLE with task-specific parameter optimization (threshold, initial sensitivity/specificity, convergence criteria) already achieved statistically significant improvements across both datasets, we employed the standard formulation. Exploring whether these STAPLE variants could yield additional gains represents a promising direction for future research. Notably, the BraTS 2024 challenge employed STAPLE to fuse segmentations from multiple neural networks during its ground truth annotation pipeline (34), demonstrating its continued relevance in large-scale medical image segmentation research. Our results suggest that STAPLE provides an effective balance between segmentation accuracy improvement and practical deployment considerations, particularly for challenging tumor segmentation tasks.

Our study has several limitations that warrant consideration. First, the ensemble approach requires substantial computational resources—training five models with different loss functions using 5-fold cross-validation demanded 25 times the computational budget compared to training a single model on the full dataset. This computational burden may limit practical adoption in resource-constrained settings. Second, while we demonstrated transferability between kidney and liver tumors, evaluation on non-abdominal organs and different imaging modalities (MRI, PET) would provide broader validation. Third, although we successfully induced model diversity through loss functions, alternative strategies such as different network architectures or training data variations might yield different or complementary benefits.

Despite these limitations, our study provides evidence that probabilistic ensemble methods can meaningfully improve tumor segmentation performance. The consistent improvements across kidney and liver tumors, combined with reduced prediction variance, suggest that STAPLE offers a practical enhancement to existing tumor segmentation workflows. As medical imaging increasingly relies on automated analysis, methods that improve both accuracy and reliability while maintaining computational feasibility will be essential for clinical translation.

## 5 Conclusion

In this study, we evaluated the STAPLE algorithm for tumor segmentation from CT images, comparing it with majority voting and soft voting ensemble methods across two organ systems. For kidney tumor segmentation on the KiTS19 dataset, STAPLE achieved a DSC of 0.74 ± 0.23, a 4.2% improvement over soft voting, with a clear performance hierarchy: STAPLE > soft voting > majority voting. These improvements remained statistically significant after Benjamini–Hochberg correction for multiple comparisons. Application to liver tumor segmentation (LiTS17) showed similar gains, with DSC of 0.76 ± 0.10 representing a 7.0% improvement and reduced variance. Stratified analysis by tumor volume revealed that STAPLE’s advantage was most pronounced for medium-sized tumors, reducing performance variability by 45%, while small tumors remained challenging across all methods. By probabilistically weighting predictions from models trained with different loss functions, STAPLE provided more consistent segmentation performance than equal-weight averaging. These improvements across kidney and liver tumors demonstrate that probabilistic ensemble methods can effectively enhance tumor segmentation accuracy across different anatomical contexts.

## Data Availability

The original contributions presented in the study are included in the article/supplementary material. Further inquiries can be directed to the corresponding author.
